# Factors affecting pregnancy registration in India: does the pregnancy intention matter?

**DOI:** 10.1186/s12884-023-06002-9

**Published:** 2023-09-19

**Authors:** Sourav Mondal, Abhishek Anand, Niharika Awasthi, Bharti Singh, Manas Ranjan Pradhan

**Affiliations:** 1 International Institute for Population Sciences (IIPS), Govandi Station Road, Deonar, Mumbai, Maharashtra 400088 India; 2 Department of Fertility and Social Demography, International Institute for Population Sciences (IIPS), Govandi Station Road, Deonar, Mumbai, Maharashtra 400088 India

**Keywords:** Pregnancy intention, Pregnancy registration, Correlates, India

## Abstract

**Background:**

Pregnancy registration is one of the most critical components of women’s reproductive health because it is the gateway to entering the continuum of care services such as antenatal care, institutional delivery, and postnatal care. There is a lack of studies exploring the relationship between pregnancy intention and pregnancy registration, especially in the Indian context.

**Method:**

This study used the National Family Health Survey-5 (2019-21) data to explore the relationship between birth intention and failure of pregnancy registration. The bivariate and multivariate (binary logistic regression) analysis was carried out.

**Results:**

Adjusting the effects of socio-demographic and economic characteristics, compared with women with an intended pregnancy, the odds of failure of pregnancy registration were significantly high among women with a mistimed pregnancy (OR = 1.60, 95% CI = 1.47–1.73) and unwanted pregnancy (OR = 1.38, 95% CI = 1.26–1.52). The study found pregnancy intention as a significant predictor of pregnancy registration.

**Conclusions:**

Results suggest strengthening the interaction of grassroots-level health workers with women, especially those with possibly lower healthcare autonomy and unintended pregnancy. Higher and earlier pregnancy registration will enhance maternal healthcare utilization and reduce adverse health consequences to mothers and children, thus ensuring better maternal and child health.

## Introduction

Maternal mortality continues to be high in India, although remarkable improvements have recently been noticed [1]. With a prevalence of 120 per 1000 live births, severe maternal morbidities indicate the equally bad maternal health status of Indian women [2]. Antenatal care (ANC), institutional delivery, and appropriate postnatal care (PNC) are the deterrent factors of maternal morbidity and mortality [3, 4]. An early ANC visit allows for the most effective screening and tests, including correct gestational age determination for proper preterm labor treatment, genetic and congenital diseases screening, folic acid supplementation to lower iron deficiency anemia, and sexually transmitted infections screening and treatment [5]. It is thus critical to identify and register women early in their pregnancies to ensure the utilization of suggested ANC services [6]. Early pregnancy registration is the starting point of Indian government’s maternal healthcare delivery system [7]. Delays in pregnancy registration influence obtaining appropriate treatment, immunization, and vitamin supplementation, all critical in reducing pregnancy-related problems. Policy initiatives to increase pregnancy registration are well in place universally in India. Pregnant women are given Mother and Child Protection (MCP) Cards and safe motherhood booklets [7], and some states have added initiatives along with this MCP card to increase pregnancy registration. For example, Karnataka has introduced the Thayi card and the mother and child tracking systems (MCTS). Pregnant women get the Thayi card with a unique identification number when registered with a female health assistant in their area, enabling socioeconomically disadvantaged women to avail of ANC and delivery services from registered private hospitals free of cost [8]. Similarly, Tamil Nadu has a Pregnancy and Infant Cohort Monitoring and Evaluation System (PICME) both in urban and rural areas [9].

Women in low-and-middle-income countries disguise their pregnancy during the first few months due to various socio-cultural behaviors and attitudes that make early pregnancy registration difficult [10, 11]. According to a study in Maharashtra, fear of losing the baby owing to black magic and the casting of evil eyes by jealous neighbors and those with malicious intentions are the causes of delayed disclosure and registration of pregnancy [12]. Women are again reluctant to inform the grassroot level health workers i.e., Accredited Social Health Activist (ASHA) and Anganwadi Worker (AWW) about their pregnancy at an early stage, as it would require them to attend the village health facility and thus, make their pregnancy evident to everyone, which they did not want [12]. ASHA, a selected trained female member from the community, works as an interface between the community and the public health system. AWW, also known as Integrated Child Development Services (ICDS) workers, are grassroots workers within the Integrated Child Development Services scheme, which aims to meet the essential health and nutritional needs of children, adolescent girls, and lactating mothers. The ASHA and AWW are the first contact points for health care seeking, mainly for women living in rural areas. Impolite health staff who do not respect confidentiality are barriers to early ANC utilization [10]. Literature reveals that women’s education is critical in obtaining early pregnancy registration [13, 14]. The average gestational age at registration and first-trimester appointment is closely connected for women with lower parity and those that have previously experienced stillbirths [13]. However, some studies show no link between a previous unfavorable obstetric history and the length of pregnancy at registration [15]. Morbidity in the index pregnancy and nulliparity favor early appointment considerably [15]. Despite considering pregnancy registration in the first trimester important, many women do not practice it, especially with increasing parity [16]. A 2009 study found that women with unplanned or mistimed pregnancies were more likely to delay starting prenatal care until after the first trimester than planned pregnancies [17]. The role of the husband behind the failure of pregnancy registration also become crucial as women are often dependent on their husband’s decisions [12]. A recent study found that socially marginalized groups, i.e., Scheduled Caste (SC), Scheduled Tribe (ST), and Other Backward Classes (OBC) have taken a longer time and late initiated the first ANC check-up (registration of pregnancy) than Non-SC/ST/OBC [18]. Most tribal women from East Khasi Hills failed to receive full ANC because they did not register early enough [19]. This late enrollment in the first ANC services among the lower caste groups might result in high infant and maternal morbidity and mortality [20]. One of the main causes of India’s unequal access to healthcare, low utilization of health services, and poor health outcomes are caste-based exclusion and discrimination [21, 22].

Early pregnancy registration contributes to improved maternal and child health. However, to our knowledge, no empirical study using nationally representative data has explored the correlates of pregnancy registration in India. Moreover, the relationship between pregnancy intention of last birth and pregnancy registration is inadequately explored. This study assesses the determinants of pregnancy registration, especially the role of the intention of the last birth in the country.

## Materials and methods

### Data

**Fig. 1:**
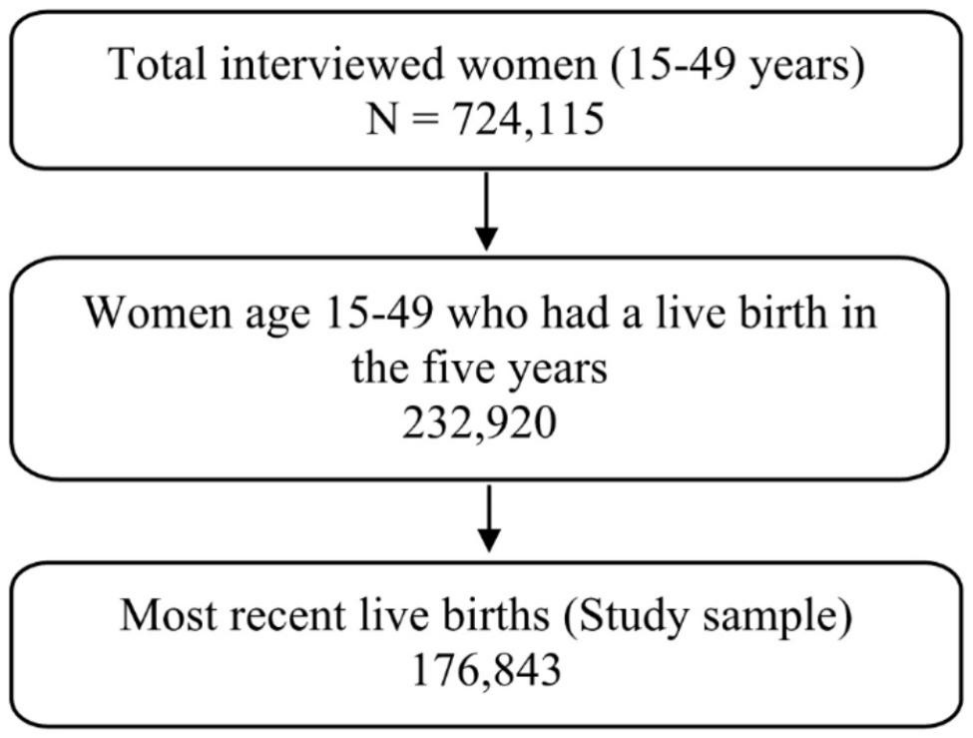
Sample selection criteria

The present study used data from the nationally representative fifth round of the National Family Health Survey (NFHS) conducted in 2019-21. The NFHS-5 provides state and district-level estimates on various indicators such as maternal and child health, fertility, mortality, nutrition, family planning, domestic violence, and women empowerment. The survey used a two-stage stratified sampling technique in all states and union territories, covering 636,699 households, 724,115 women aged 15–49, and 101,839 men aged 15–54. The response rates of women, men, and households in NFHS-5 were 97%, 92%, and 98%, respectively. An informed consent procedure was followed, and only those who voluntarily consented were interviewed. The survey report contains the survey protocol, sampling, data collection tools, and quality control measures [23]. The survey has collected the pregnancy registration information for the most recent live birth to women during the five years preceding the survey. Specifically, the sample of women for whom the pregnancy registration status (N = 176,843) was available was analyzed for this study (Fig. 1).

### Outcome variable

The outcome variable used in this analysis was ‘failure of pregnancy registration.‘ In the survey, for the last birth child, mothers were asked, ‘Was this pregnancy registered?’. Based on the response from mothers, the variable was coded in 0 as “no” and 1 as “yes.“

### Exposure variables

This study included relevant exposure variables suggested by existing literature [15, 17]. Pregnancy intention of last birth was our main exposure variable, categorized into wanted, mistimed, and unwanted pregnancy. Additionally, the other key variables included in the analysis were the age of women (15–19, 20–24, 25–29, 30–34, 35–39 y, 40–49), years of schooling of the women (no schooling, 1–5 years, 6–10 years, 10 + years), parity (1, 2–3, 4+), history of obstetric complications (no, yes), interaction with ASHA)/AWW in last three months (no, yes), mass media exposure (exposed, not exposed), religion (Hindu, Muslim, others), social group (SC, ST, OBC, others), wealth index of the household (poorer, poor, middle, richer, richest), place of residence (rural, urban) and geographical region (north, central, east, northeast, west, south). Women exposed to television, radio, newspapers, and cinema were considered mass media exposure.

### Statistical methods

We used bivariate and multivariable analysis to analyze the data. We used a logistic regression model to examine the association between exposure and outcome variables which were dichotomous.

$$logit\,p=ln\left(\frac{p}{1-p}\right)$$

$$={b}_{0}+{b}_{1}{x}_{1}+{b}_{2}{x}_{2}+\dots +{b}_{n}{x}_{n}e$$

Where ${b}_{0}, {b}_{1}, {b}_{2},.,{b}_{n}$are coefficients of each exposure variable and ‘*e*’ denotes the error term. Binary logistic regression was conducted to examine the adjusted effect of socio-economic and demographic predictors of pregnancy registration and registration in the first trimester. The predictor variables included in the regression analysis were finalized after assessing their independent association with the outcome variable (pregnancy registration) and checking collinearity among the predictor variables. Multicollinearity was assessed through the Variance Inflation Factor (VIF) method. National individual sample weight was used in the analysis. The analysis was done in Stata (Version 16) with a 5% significance level.

## Results

**Fig. 2:**
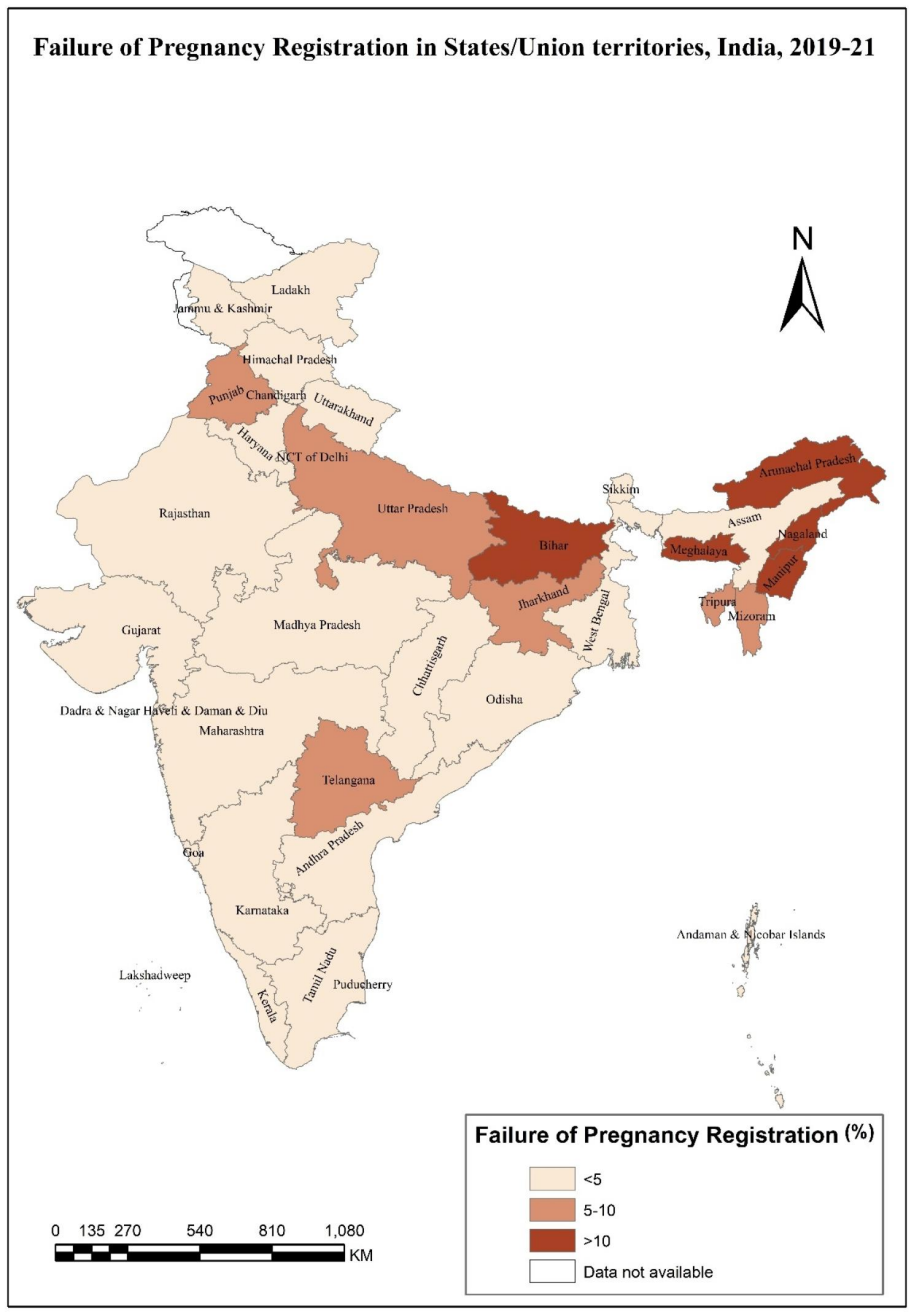
Failure of pregnancy registration in States/Union Territories, India, 2019-21

Table 1 shows the prevalence of failure of pregnancy registration by women’s socio-economic, demographic, and health-related characteristics. In 2019-21, 6% of the women did not register their last pregnancy. Women with mistimed pregnancies (12.6%) had the maximum registration failure, followed by unwanted pregnancies (8.2%). Failure of pregnancy registration had increased with the age of women. The highest proportion of failure of pregnancy registration was among women aged 40–49 years (11.2%). 8% of Muslim women failed to register their pregnancy compared to women who belong to other religions (6.6%) and Hindu women (5.8%). Women with no education and belonging to the lowest wealth quintile had the highest prevalence of failure of pregnancy registration (10.6% and 9.4%, respectively). The failure of pregnancy registration among women with four and above parity was twice as much as for women with one parity. Women who had consulted the ASHA/AWW in the last three months had the lowest prevalence (2.9%). Similarly, women exposed to any media had the lowest prevalence (4.9%). Failure in pregnancy registration also showed regional variation, southern India had the lowest, and the eastern part had the highest prevalence of failure of pregnancy registration. By looking at states/union territories, Nagaland (32%) had the highest percentage of failure to register pregnancy, followed by Bihar (16%), Manipur (15%), and Arunachal Pradesh (14%). The lowest percentage of failure to register pregnancy was found in Odisha, Karnataka, and Tamil Nadu, and all of these states had a 2% failure in pregnancy registration (Fig. 2).

**Table 1:** Failure of pregnancy registration by women’s socio-economic, demographic, and health-related characteristics, India, 2019-21

Exposure Variables	Failure of pregnancy registration	
	%	Total
**Pregnancy Intention of Last Birth**		
Wanted	5.8	1,62,709
Mistimed	12.6	6,980
Unwanted	8.2	7,155
**Age Group**		
15–19	5.1	5,510
20–24	5.4	51,723
25–29	5.7	68,532
30–34	6.8	34,757
35–39	8.3	12,772
40–49	11.4	3,549
**Place of Residence**		
Urban	6.4	49,876
Rural	6.1	1,26,967
**Religion**		
Hindu	5.8	1,40,715
Muslim	7.6	28,145
Others	6.6	7,983
**Caste**		
SC	6.2	40,057
ST	5.7	17,479
OBC	6.2	76,047
Others	6.1	43,260
**Years of Schooling**		
No schooling	10.6	34,722
1–5 years	6	20,541
6–10 years	4.4	65,984
10 + years	5.4	55,597
**Wealth Index**		
Poorer	9.4	40,270
Poor	5.8	37,210
Middle	4.4	34,620
Richer	4.4	34,015
Richest	6.2	30,728
**Parity**		
1	4.9	60,612
2–3	5.7	93,363
4+	11.2	22,868
**History of Obstetric Complications**		
No	6.2	1,57,709
Yes	5.8	19,134
**Consulted ASHS/Anganwadi workers in last 3 months**		
No	13.3	55,077
Yes	2.9	1,21,766
**Mass Media Exposure**		
Not Exposed	9.6	46,481
Exposed	4.9	1,30,362
**Region**		
North	4.4	24,027
Central	7.1	47,130
East	9.3	45,618
Northeast	5.4	7,168
South	2.9	30,045
West	4.1	22,854
**Total**	**6.1**	**1,76,843**

Table 2 presents the result of the logistic regression of factors affecting the pregnancy registration. Model 1 shows the unadjusted odds ratio (OR). Women with mistimed pregnancies (OR = 2.26, 95% CI = 2.09–2.44), and unwanted pregnancies (OR = 1.42, 95% CI = 1.30–1.55) had higher odds of not registering their pregnancy as compared to women with wanted pregnancies. This association remained significant after adjusting the effects of other socio-economic and demographic characteristics used in Model 2. Mistimed and unwanted pregnancies had 60% (OR = 1.60, 95% CI = 1.47–1.73) and 38% (OR = 1.38, 95% CI = 1.26–1.52) higher likelihood of non-registration. Age had no statistically significant association with the failure of pregnancy registration. Women with higher education and wealth status were less likely to fail in registering their pregnancy than those with no schooling and the poorest wealth status. Women belonging to SC and OBC had a weak association with pregnancy registration. As compared to Hindu women, Muslim women (OR = 1.10; 95% CI = 1.04–1.16) and women who follow other religions (OR = 1.44; 95% CI = 1.30-1. 59) had a higher likelihood of failing in pregnancy registration. The parity of the women was directly proportional to the failure of pregnancy registration. Higher the parity of women, the higher the odds of failure of pregnancy registration. Women who consulted the ASHA/AWW in the last three months had a 79% (OR = 0.21; 95% CI = 0.20–0.22) lower chance of failure of pregnancy registration. Likewise, women exposed to mass media had lower odds of failure in pregnancy registration. Considering the regional variation, women of central (OR = 1.94; 95% CI = 1.79–2.11), eastern (OR = 2.24; 95% CI = 2.06–2.44), and north-eastern (OR = 1.26;95% CI = 1.10–1.44) part of the country had a significantly higher likelihood of failure of pregnancy registration compared to women of the southern region.

**Table 2:** Logistic regression to examine the association between failure of pregnancy registration by women’s socio-economic, demographic, and health-related characteristics, India, 2019-21

Exposure Variables	Failure of Pregnancy Registration			
	Model 1		Model 2	
	UOR	95% CI	OR	95% CI
**Pregnancy Intention of Last Birth**				
Wanted®	1	[1.00,1.00]	1	[1.00,1.00]
Mistimed	2.26***	[2.09,2.44]	1.60***	[1.47,1.73]
Unwanted	1.42***	[1.30,1.55]	1.38***	[1.26,1.52]
**Age Group**				
15–19®			1	[1.00,1.00]
20–24			1.08	[0.95,1.24]
25–29			1.04	[0.91,1.19]
30–34			1.06	[0.92,1.22]
35–39			1.1	[0.95,1.28]
40–49			1.16	[0.97,1.39]
**Years of Schooling**				
No schooling®			1	[1.00,1.00]
1–5 years			0.73***	[0.68,0.78]
6–10 years			0.66***	[0.62,0.70]
10 + years			0.83***	[0.78,0.89]
**Parity**				
1®			1	[1.00,1.00]
2–3			1.09***	[1.04,1.15]
4+			1.45***	[1.34,1.57]
**History of Obstetric Complications**				
No®			1	[1.00,1.00]
Yes			0.98	[0.92,1.05]
**Consulted ASHS/Anganwadi workers in last 3 months**				
No®			1	[1.00,1.00]
Yes			0.21***	[0.20,0.22]
**Mass Media Exposure**				
Not exposed			1	[1.00,1.00]
Exposed			0.77***	[0.73,0.81]
**Caste**				
SC			1.02	[0.96,1.09]
ST			0.96	[0.88,1.05]
OBC			1.06*	[1.00,1.12]
Others®			1	[1.00,1.00]
**Religion**				
Hindu®			1	[1.00,1.00]
Muslim			1.10***	[1.04,1.16]
Others			1.44***	[1.30,1.59]
**Wealth Index**				
Poorer®			1	[1.00,1.00]
Poor			0.82***	[0.78,0.88]
Middle			0.76***	[0.70,0.81]
Richer			0.78***	[0.72,0.85]
Richest			0.99	[0.90,1.08]
**Place of Residence**				
Urban®			1	[1.00,1.00]
Rural			0.87***	[0.82,0.91]
**Region**				
South®			1	[1.00,1.00]
North			1.09	[0.99,1.20]
Central			1.94***	[1.79,2.11]
East			2.24***	[2.06,2.44]
Northeast			1.26***	[1.10,1.44]
West			0.92	[0.84,1.02]
**Observations**	**176,843**			

® “Reference category”, * “p < 0.05”, ** “p < 0.01”, *** “p < 0.001

## Discussion

The study found that pregnancy registration is not universal in India. The determinants of pregnancy registration varied considerably by socio-economic and demographic characteristics plus pregnancy intention of the last birth. The pregnancy intention of the last birth had a robust association with the pregnancy registration. Maternal education, parity, mass-media exposure, interaction with health workers, household wealth index, social group, religion, and region were other significant determinants of pregnancy registration.

The pregnancy intention of the last child is a significant determinant of the pregnancy registration. The failure of pregnancy registration was higher among women with either a mistimed or an unwanted last child. This result is in line with the previous studies, which found that unplanned birth had less chance of pregnancy registration and other negative implications on women’s reproductive and mental health [17, 24]. Non-registered pregnancies are again less likely to receive appropriate ANC, thus putting women at higher risk of maternal complications. Earlier studies reveal that women with inadequate ANC had higher maternal morbidities and mortality [25]. There is also low institutional delivery for those unintended births [26]. Additionally, inadequate ANC and non-institutionalized births for unintended pregnancies are detrimental to child health [27]. Early pregnancy registration would ensure appropriate ANC, vaccination, supplementary foods from ICDS, institutional delivery, and PNC, thus improving maternal and child health.

Maternal education positively influences the utilization of various maternal healthcare services [28]. Our study also found that educated women are more likely to register their pregnancies. As evidenced in a past study [15], this study also found an inverse association between failure to register pregnancy and parity. An African study revealed that women with earlier births cited their experience identifying maternal complications and frequent travel for ANC as the reason for delayed or no registration. We found that women with a history of pregnancy complications are less likely to fail to register their pregnancy. This conforms with earlier studies in India [14] and Africa [15]. The study found that interaction with the ASHA/AWW is beneficial for pregnancy registration. An earlier study [29] also documented the beneficial role of ASHA/AWW in enhancing the utilization of maternal healthcare services, including pregnancy registration. Nevertheless, some past studies suggest that due to impolite behavior and lack of confidentiality, women do not want to inform the ASHA and AWW about their pregnancy at an early stage, which lead to non-registration of pregnancy [10, 12]. Mass media exposure was found beneficial so far as pregnancy registration is concerned. This result conforms with a past study that reveals its contribution to the utilization of maternal healthcare services [30]. As compared to Hindu women, Muslim women and women who follow other religions are less likely to register their pregnancy. A previous study confirms the critical role of religion as a key determinant of women’s reproductive health-seeking behavior, as different religions follow different beliefs [31]. Past studies found wealth status of the household positively influences health-seeking behavior [20, 32]. Our study also reveals that failure of pregnancy registration decreases with the increasing economic condition of the household. Higher chances of pregnancy registration in rural areas may be credited to the outreach of the grass-root level health workers like ASHA/AWW. There is enough literature to prove the significant contribution of ASHA/AWW to improved maternal health [29, 33]. This study found a wide regional disparity in the registration of pregnancy. The lower likelihood of pregnancy registration in the country’s central and eastern regions, which comprise a sizable proportion of the country’s reproductive-age women, is a cause of concern.

Results suggest provision of context-specific, and culturally sensitive awareness programs regarding the benefits of early pregnancy registration to all eligible couples preferably by local grassroots-level health workers (ASHAs and AWWs). The risks of late pregnancy registration and the benefits of early pregnancy registration can also be advocated through several public interaction initiatives, i.e., awareness campaigns, street play, quizzes, lectures by local administrative authorities, such as Panchayati Raj Institutions (PRI), local educational institutions, and non-government organizations (NGO). Some previous studies have also advocated this [12, 14].

### Strengths and limitations of the study

There are several strengths and limitations of this study. The findings are based on a large nationally representative sample of women covered in the NFHS-5 with a robust sampling design, which makes the findings relevant for policy and program. Secondly, to our knowledge, this is the first study to investigate several socio-economic and demographic determinants of pregnancy registration at a national level. However, the survey’s cross-sectional design limits pregnancy registration’s causal association with factors drawn from this analysis. Additionally, other socio-cultural factors could influence pregnancy registration, which could not be included in this analysis due to their unavailability in NFHS data. Despite this, the study’s findings would help strengthen the existing service delivery mechanism to increase early and overall pregnancy registration, thus ensuring better maternal and child health in the country.

## Conclusion

Pregnancy intention is significantly associated with pregnancy registration in India. The grassroots-level health workers may strengthen their efforts to highlight the benefits of pregnancy registration, especially among women with possibly lower healthcare autonomy and unintended pregnancy. Pregnancy registration will enhance maternal healthcare utilization and reduce adverse health consequences for mothers and children, thus ensuring better health.

## Data Availability

The datasets generated and/or analysed during the current study are available in the [Demographic and Health Surveys Repository] repository, [https://dhsprogram.com].
